# Efficacy of clozapine versus standard treatment in adult individuals with intellectual disability and treatment-resistant psychosis (CLOZAID): study protocol of a multicenter randomized clinical trial

**DOI:** 10.3389/fpsyt.2024.1400621

**Published:** 2024-05-14

**Authors:** María Alemany-Navarro, Bianca Sánchez-Barbero, Pablo Reguera-Pozuelo, Laura Altea-Manzano, Ana Gómez-Garrido, Idalino Rocha-González, Nathalia Garrido-Torres, Miguel Ruiz-Veguilla, Susana García-Cerro, Clara M. Rosso-Fernández, José María Villagrán-Moreno, Fernando Sarramea, Jorge Cervilla-Ballesteros, Rafael Martínez-Leal, Fermín Mayoral-Cleries, CLOZ-AID Group, Samuel Romero Guillena, Benedicto Crespo-Facorro

**Affiliations:** ^1^ Translational Psychiatry Group, Seville Biomedical Research Institute (IBiS)-CSIC, Seville, Spain; ^2^ Foundation for Health Research Management in Sevilla, Sevilla, Spain; ^3^ Spanish Network for Biomedical Research in Mental Health (CIBERSAM), Madrid, Spain; ^4^ Mental Health Unit, Virgen del Rocio University Hospital, Seville, Spain; ^5^ Department of Psychiatry, Faculty of Medicine, University of Seville, Seville, Spain; ^6^ Clinical Research and Clinical Trials Unit (CTU), Virgen del Rocío Hospital, Seville, Spain; ^7^ Mental Health Unit, Área de Gestión Sanitaria Jerez, Costa Noroeste y Sierra de Cádiz, Cádiz, Spain; ^8^ Neurosciences Department, University of Cádiz, Cádiz, Spain; ^9^ Instituto de Investigación e Innovación Biomédica de Cádiz (INiBICA), Cádiz, Spain; ^10^ Área de Gestión Sanitaria Córdoba, Córdoba, Spain; ^11^ Hospital Universitario San Cecilio, Granada, Spain; ^12^ Psychiatry Department, University of Granada, Granada, Spain; ^13^ Fundació Villablanca, Unidad de Investigación en Discapacidad Intelectual y Trastornos del Desarrollo (UNIVIDD), Institut d'Investigació Sanitària Pere Virgili (IISPV), Reus, Spain; ^14^ Psychology Department, Universitat Rovira i Virgili, Tarragona, Spain; ^15^ Psychiatry Unit, Hospital Regional Universitario de Málaga, Málaga, Spain

**Keywords:** clozapine, intellectual disability, resistant psychosis, effectiveness, antipsychotics

## Abstract

**Background:**

Intellectual disability (ID) affects approximately 1% of the worldwide population and individuals with ID have a higher comorbidity with mental illness, and specifically psychotic disorders. Unfortunately, among individuals with ID, limited research has been conducted since ID individuals are usually excluded from mental illness epidemiological studies and clinical trials. Here we perform a clinical trial to investigate the effectiveness of clozapine in the treatment of resistant psychosis in individuals with ID. The article highlights the complexity of diagnosing and treating psychopathological alterations associated with ID and advocates for more rigorous research in this field.

**Methods:**

A Phase IIB, open-label, randomized, multicenter clinical trial (NCT04529226) is currently ongoing to assess the efficacy of oral clozapine in individuals diagnosed with ID and suffering from treatment-resistant psychosis. We aim to recruit one-hundred and fourteen individuals (N=114) with ID and resistant psychosis, who will be randomized to TAU (*treatment as usual*) and treatment-with-clozapine conditions. As secondary outcomes, changes in other clinical scales (PANSS and SANS) and the improvement in functionality, assessed through changes in the Euro-QoL-5D-5L were assessed. The main outcome variables will be analyzed using generalized linear mixed models (GLMM), assessing the effects of status variable (TAU vs. Clozapine), time, and the interaction between them.

**Discussion:**

The treatment of resistant psychosis among ID individuals must be directed by empirically supported research. CLOZAID clinical trial may provide relevant information about clinical guidelines to optimally treat adults with ID and treatment-resistant psychosis and the benefits and risks of an early use of clozapine in this underrepresented population in clinical trials.

**Trial registration:**

Clinicaltrials.gov: NCT04529226. EudraCT: 2020-000091-37.

## Background

Intellectual disability (ID) is defined as the existence of significant limitations both in intellectual functioning and adaptive behavior, including restrictions in conceptual, social, and practical skills (1). ID originates before the age of 18 (2) and has a prevalence of approximately 1%, with a breakdown of 0.37-0.59% for mild intellectual disability and 0.3-0.4% for moderate, severe, and profound intellectual disability combined (3).

Individuals with ID constitute a highly vulnerable group, presenting a lower life expectancy and greater comorbidity, which is reflected in higher care needs on the part of the health system (4) and the consequent greater health system and family expenditure compared to those without ID (5–7). Remarkably, individuals with ID present greater prevalence of mental illnesses, with a co-diagnosis of mental illness and ID in approximately 30-50% of cases (8, 9). Particularly, studies indicate that the lifetime prevalence of psychotic disorders in individuals with ID ranges from 0.4% to 7.8% (8, 10, 11), while the prevalence of schizophrenia in the general population is at 0.8% (12, 13). The prevalence of psychotic disorders is higher in moderate ID, reaching 6%, while it is lower in mild and deep ID (3.5%) (8). Despite mental illness being underdiagnosed in up to 30% of cases involving individuals with ID (14), this is primarily due to the challenges associated with clinical evaluation, particularly in cases of moderate to severe ID (15, 16).

Frequently, we witness either overmedication or an inappropriate prescription regimen for certain drugs in the treatment of mental illness, leading patients into a diagnostic and therapeutic cascade. This is attributed to the fact that nearly every drug is associated with side effects, especially in individuals who are particularly sensitive, such as those with ID (17), for who pharmacodynamics (absorption, distribution, metabolism and excretion) may be altered (18).

Among antipsychotics, clozapine is the most effective one for individuals with non-affective psychosis who do not respond to other first- and second-generation antipsychotic treatments (19, 20). Clozapine has proved to be very effective in other kinds of clinical situations as well, such as hostility and aggressiveness, polydipsia, and behavioral disorders, all of them frequent situations in individuals with ID (21–24), for who antipsychotics are the most prescribed drugs (18). Additionally, clozapine has been associated with a decrease in hospital admissions and the number of days of hospitalization (25).

Despite of, clozapine is clearly underused in clinical practice due to a series of barriers and difficulties in its effects and clinical management (26). Although very infrequently, clozapine is associated with several side effects (27). Some of them are myocarditis and cardiomyopathy during initial phases of treatment (28), seizures (29), and neutropenia (30). The risk of severe neutropenia has led to the mandatory monitoring of the number of neutrophils in blood regularly during the treatment. Constipation is another possible adverse effect associated with clozapine, which can lead to be serious and have fatal consequences, with complications such as paralytic ileus or Bowel perforation (31). Therefore, the decision to introduce clozapine should always stem from a meticulous risk-benefit analysis due to potential complications.

The early utilization of clozapine in individuals resistant to other antipsychotics and in specific clinical scenarios, where clozapine has demonstrated efficacy, presents an opportunity for improvement and evolutionary stabilization for those individuals based on the clinical remission or recovery produced (32). Enhancing access to clozapine could bring about clinical benefits for numerous individuals, coupled with evident economic savings within the healthcare system (33, 34). Furthermore, by improving access to clozapine, the risk associated with polypharmacy (simultaneous use of multiple antipsychotics) in the absence of clinical response could be mitigated, thereby reducing the chances of overdosage and ineffective treatment. Lastly, considering that the appearance of resistant psychotic symptoms and associated behavioral disorders in this population represents an added stress factor to the burden chronic disability for both patients and their caregivers, expected clinical benefits of clozapine treatment appear to outweigh the potential physical risks associated with treatment.

In a recent Cochrane review with the objective of reviewing the effectiveness of clozapine and its adverse side effects in the treatment of individuals with the dual diagnosis of psychosis and ID, it is concluded that there are only descriptions of its effectiveness in individual cases or in groups of patients with a very small number, with no evidence yet available from randomized clinical trials of said effectiveness (efficacy and safety) (35). Only a few studies have been carried out on it, since individuals with ID are usually excluded from epidemiological studies of mental illness and especially from clinical trials (15, 16, 35).

In this way, the use of clozapine in individuals with ID and resistant psychosis is based on the extrapolation of evidence in the general population without ID suffering from schizophrenia resistant to antipsychotic treatment (36). Therefore, it is crucial to conduct randomized and controlled clinical trials involving a population with ID experiencing a high prevalence of resistant psychosis to conventional antipsychotic treatments. However, to minimize possible complications, developing these trials requires a detailed and rigorous study of the safety profile of the drug in this vulnerable population.

The high complexity in the diagnosis (difficulty of these patients in describing the symptoms) and the treatment of behavioral and psychopathological alterations associated with ID has resulted in a scarcity of services and research in this domain (37). It is desirable to carry out a more extensive number of research studies with methodological rigor to provide substantial evidence about this significant public health issue in a particularly vulnerable population. Hence, in the present article we introduce the study protocol conducted in the CLOZAID randomized controlled clinical trial.

## Methods

### Study design

The CLOZAID trial (NCT04529226) is a prospective, randomized, flexible-dose, open-label, phase IIB clinical trial designed to assess the efficacy of oral clozapine in individuals diagnosed with ID and suffering from treatment-resistant psychosis. This trial aims to assess and compare the effectiveness of clozapine in managing treatment-resistant psychosis in individuals with ID against the outcomes observed in the usual clinical practice, potentially contributing to enhanced treatment strategies and improved outcomes for this challenging patient population. Treatment resistance was determined according to Howes et al. (38) criteria, considering the chlorpromazine equivalents (CPZeq) proposed by Woods (2003) (39): olanzapine 5–20 mg/day (100–400 CPZeq), risperidone 3–6 mg/day (150–300 CPZeq), haloperidol 3–9 mg/day (150–450 CPZeq), quetiapine 100–600 mg/day (133.33–800 CPZeq), ziprasidone 40–160 mg/day (66.67–266.67 CPZeq) and aripiprazole 5–30 mg/day (66.67–400 CPZeq).

The patient follow-up in this study consists of 6 scheduled visits. The procedures carried out at each visit can be seen in **Table 1**.

**Table 1:** Assessment conducted during 6 scheduled clinical visits.

	Month 0	Month 1	Month 1 ± 10 days	Month 3 ± 15 days	Month 6 ± 30 days	Month 12 ± 30 days	
	Visit 1 Screening	Visit 2 Randomization (Day 1 of treatment)	Visit 3	Visit 4	Visit 5	Visit 6	Unscheduled visit*
Informed consent/ACE	●						
Inclusion/exclusion criteria	●	●				●	
Pregnancy test	●	●				●	
Medical history	●	●	●	●	●	●	
Physical exploration**^1^**	●	●	●	●	●	●	●
Functioning: **POMONA II, ICAP**		●					
Clinical improvement scales: **GCI, PANSS, SANS**		●	●	●	●	●	●
Adherence to treatment: **Morisky-Green Test** and **BARS** (1)		●	●	●	●	●	●
Cognitive tests: **K-BIT**	●					●	●
Psychopathology, functioning and aggressiveness tests: **MINI PAS-ADD** **EURO-QoL-5D-5, ABC**, among others		●	●	●	●	●	●
Suicide test: **C-SSRS**	●	●	●	●	●	●	●
Hematology/biochemistry**^2^**	●	●	●	●	●	●	●
Alcohol/drug use		●	●	●	●	●	●
Concomitant medication	●	●	●	●	●	●	●
Treatment		●	●	●	●	●	●
Adverse events**^3^**: **SAS, BARS** (2)			●	●	●	●	●

*Unscheduled visit. If a patient shows significant changes in their clinical condition or if there is suspicion of an adverse event, an assessment (within 24 hours) should be conducted by phone to determine the necessity of an unscheduled visit; ACE, Aid to Capacity Evaluation; POMONA, Health Indicators for People with Disabilities; ICAP, Inventory for Client and Agency Planning; GCI, Global Clinical Impression; PANSS, Positive and Negative Syndrome Scale; SANS, Scale for the Assessment of Negative Symptoms; BARS (1), Brief Adherence Rating Scale; K-BIT, Kaufman Brief Intelligence Test; MINI PAS, Psychiatric Assessment Schedule for Adults with Developmental Disability; ABC, Aberrant Behavior Checklist; C-SSRS, Columbia-Suicide Severity Rating; SAS, Simpson-Angus Scale; BARS (2), Barnes Akathisia Rating Scale.

^**1.**^Includes weight, height, blood pressure, heart rate, respiratory rate, temperature, and abdominal circumference.

^**2.**^Main attention to lipid and glycemic profile; blood count control (special leukocytes/neutrophils control).

^**3.**^Including hospital admissions, seizure/dizziness, constipation.

### Study setting

CLOZAID is a multicenter study that is being conducted at 25 clinical centers throughout Spain (**Supplementary Table 1**).

### Individual recruitment and eligibility criteria

Recruitment strategies will include providing information to individuals during consultations, as well as conducting a thorough search across different databases based on established clinical criteria. Individuals who accept to participate in the trial will be screened and their diagnosis will be confirmed by experienced psychiatrists through the clinician version of the structured clinical interview for the Diagnostic and Statistical Manual of Mental Disorders, Fifth Edition (DSM-5) (SCID-5-CV; 40) at each center. Written informed consent will be gathered before being enrolled in the trial. No restrictions will be enforced on the source of the individuals (outpatients or inpatients). **Table 2** shows the different eligibility criteria.

**Table 2:** Eligibility criteria for participant recruitment.

Inclusion criteria	Exclusion criteria
1) Age between 16-55	1) Levels of leukocytes <3500/mm^3^ and neutrophils <2000/mm^3^
2) Main diagnosis of intelectual deficit (DSM-V) and confirmed using the brief Kaufman intelligence test (Annex V), IQ: 35-70	2) Hypersensitivity to clozapine or its excipients
3) Diagnosis of psychotic disorder (DSM-V) confirmed by clinical interview	3) History of Myeloproliferative Syndrome
4) Resistance to antypsychotic treatment (at least two different, except clozapine) at the maximum dose for a duration of treatment ≥ 6 weeks	4) Epilepsy not controlled with medication in the previous 2 years
5) Behavioral alterations, self-harm and/or severe stereotypes during the 6 months prior to inclusion	5) Paralytic ileus 3 months before inclusion
6) Consent by written to participate in the trial of patients and/or their legal representatives	6) Patients diagnosed at study entry or selection visit itself of autism spectrum disorders
7) Capacity and availability to carry out the assesments of the trial protocol or facilitated	7) Pregnant women at the time of study entry or wishing to start pregnancy within 12 months after entering the same
8) In case of potential risk of pregnancy, negative pregnancy test and/or contraception	8) Women who are breast-feeding at the time of entry into the study who do not accept its withdrawal
	9) Demonstrated medical pathology (heart disease, intestinal transit disorders…) that contraindicate the use of clozapine
	10) Any serious medical pathology not controlled at the time of entry into the hospital
	11) Treatment at the beginning of the study that cannot be withdrawn with any prohibited drug during the study
	12) Patients in whom a high risk of suicide is detected at the screening visit, according to the criteria established by Columbia University according to the evaluation of said risk using the C-SSRS scale (Annex V)
	13) Patients who are participating in another clinical trial with active treatment (1) meeting DSM-IV criteria for drug dependence; (2) meeting DSM-IV criteria for mental retardation; (3) having a history of neurological disease or head injury
Based on efficacy criteria	Based on safety criteria
1) Patients with a drug compliance level below 80%	1) Any adverse event that, at the clinician’s discretion, requires study withdrawal.
2) Patients who, at any time during the study period, show a high risk of suicide based on criteria established by Columbia University, as assessed by the C-SSRS scale.	2) When, for any reason, the treatment is no longer safe for the patient.
3) Female patients who test positive for pregnancy during their participation in the study	3) Any other reason that could endanger the patient’s life or have serious consequences for them.
4) Patients who experience a convulsive seizure during their participation in the study	**Based non-compliance or violation of the norms outlined in the protocol criteria**
5) Patients randomized to the clozapine branch who have white blood cell counts <3500/mm^3^ and neutrophil counts <2000/mm^3^ at any time during their participation in the study	1) If the patient fails to comply with the trial’s norms, they may be withdrawn at the discretion of the responsible investigator or due to loss of follow-up.
6) Patients randomized to the clozapine branch who need to start treatment with drugs known to cause agranulocytosis or bone marrow depression	**Follow-up of prematurely withdrawn patients**
7) Patients randomized to the clozapine branch who require hospitalization due to intestinal obstruction or develop paralytic ileus	1) If a patient is prematurely withdrawn from the trial, the investigator will provide the main reason for the suspension; the standard treatment protocols for their condition will be followed at the discretion of the responsible clinician.
8) Patients randomized to the clozapine branch who, after the transition period to monotherapy with CZP, require treatment with more than one antipsychotic drug, ECT, or rTMS based on clinical criteria	
9) Patients randomized to the clozapine branch who need to start treatment with medications that significantly interfere with clozapine metabolism through the cytochrome P450 pathway: macrolides, antifungals, proton pump inhibitors, and trihexyphenidyl	

### Sample size

The sample size calculation was performed using the GRANMO program, a tool for determining sample size and power of a hypothesis test. Accepting an alpha risk of 0.05 and a beta risk of 0.2 in a two-tailed test, 57 individuals were required in both the first and second groups to detect a difference equal to or greater than 0.8 units. We assumed a common standard deviation of 1.4, and a loss rate of 15%.

### Data collection

#### Randomization

We used a simple randomization procedure (1:1). An automated randomization list was drawn up. The system for randomization will be available online in the specifically designed Clinical Research Database (CRD). The randomization list will be safeguarded by the Clinical Research and Clinical Trials Unit (CRCTU). After confirming that the patient meets all inclusion criteria and none of the exclusion criteria, the investigator will proceed to administer the assigned treatment and provide the patient code, which will consist of a numeric code identifying the center and the patient (e.g., XX-XXX). There are no masking techniques as this is an open-label study. Therefore, there is no applicable procedure for breaking the blind.

Demographic and clinical characteristics, comorbidities, and psychiatric history were collected at intake, along with prior health care resource utilization and patient-reported outcomes.

#### Study Flow

Individuals with inclusion criteria diagnoses were randomized to open-label clozapine or TAU (*treatment as usual*). Treatment with clozapine began with an initial dose of 12.5 mg, gradually titrated to reach a therapeutic dose within the range of 200-450 mg. The dose and type of antipsychotic medication could be changed based on clinical efficacy and the profile of side effects during the follow-up period. Doses could be adjusted as clinically indicated within the prescribed range to target the minimum effective dose. Certain concomitant medications (lormetazepam and clonazepam) were permitted for the management of agitation, general behavior disturbances, and/or insomnia. Only if clinically significant extrapyramidal symptoms occurred, anticholinergic medication (biperiden at a dose of up to 8 mg/day) was allowed. Antidepressants and mood stabilizers were permitted if clinically needed. The doses of these concomitant medications followed standard clinical practice guidelines, and no maximum doses were specified due to the pragmatic nature of the clinical trial.

#### Measurements

##### Primary outcome measures: efficacy and effectiveness

The main outcome of efficacy and effectiveness consisted of the clinical improvement assessed with the change in the total score of the *Clinical Global Impression-Schizophrenia scale* (CGI-SCH; 41), which assesses the global severity of schizophrenia symptoms and how it impacts the individual’s overall life. A second outcome measure of effectiveness was the rate of treatment discontinuation for any reason, expressed as the percentage of individuals who discontinue the initially assigned treatment, along with the mean time to discontinuation for all causes. Four reasons for the discontinuation were recorded (1): nonsufficient or insufficient efficacy, (2) serious adverse events, (3) nonadherence, and (4) other causes. Insufficient efficacy was established at the treating physician’s judgment only after at least 12 weeks of adequate treatment after failing to reach at least a 30% reduction in the Brief Psychiatric Rating Scale. Adherence to antipsychotic drugs was assessed by the information obtained from the individuals, close relatives, and staff involved in the follow-up. Individuals were consensually dichotomized into having a good (defined as individuals regularly taking at least 90% of prescribed medication) and a poor adherence (medium or poor compliance).

##### Secondary outcome measures: efficacy and safety

Other clinical improvement variables were measured with total scores of *The Positive and Negative Syndrome Scale* (PANSS; 42) and the *Scale for the Assessment of Negative Symptoms* (SANS; 43), and improvement in quality of life and functionality assessed through changes in the Euro-QoL-5D-5L (44).

Follow-up clinical evaluations will be conducted at each center following standard clinical practice to assess the safety profile of the trial treatment:

- Physical examination: A physical examination and vital signs assessment will be performed at each visit.
- Laboratory test: Blood extraction with a complete blood count and biochemistry, including lipid profile and glucose levels, during the second visit. After the second visit, individuals on clozapine should have hematological monitoring weekly (for the first 18 weeks), then bi-weekly until month 12, and thereafter the monitoring will be monthly.

Follow-up adverse events will be monitored:

- Hematological abnormalities: Neutropenia
- Cardiological abnormalities: Myocarditis
- Severe constipation: Paralytic ileus
- Severe sedation
- Neurological abnormalities: Seizures
- Metabolic abnormalities: Diabetes mellitus, Hyperosmolar coma, Ketoacidosis, Severe hyperglycemia, Hypercholesterolemia, Hypertriglyceridemia.

The principal investigator or a collaborator is required to promptly report all serious adverse events, whether related to the treatment or anticipated, to the Virgen del Rocío University Hospital (VRUH) Pharmacovigilance Unit (FV) of the CRCTU (FV-CRCTU-VRUH) within 24 hours (one working day). Any serious adverse events occurring from the patient’s inclusion in the study (defined as the moment the subject signs the informed consent) up to 30 days after the subject completes or withdraws from the study must be reported, by completing and signing the Serious Adverse Event Report Form. The Pharmacovigilance staff will review the received form and, if necessary, request additional information from the investigator, who will provide information whenever requested and, in any case, when there is a change in their initial assessment regarding severity or causality. The procedure for reporting follow-up information will follow the same notification process. The FV-CRCTU-VRUH personnel will maintain a detailed record of all serious adverse events reported by the investigators.

If there is a medication error or if the investigational drug is used outside the protocol during the study, the investigator will notify the FV-CRCTU-VRUH within 24 hours after becoming aware of it. The notification procedure will be the same as for serious adverse events.

##### Data monitoring and quality control

A multicenter clinical monitoring has been established to ensure research quality. Prior to individual recruitment, uniform preclinical training has been conducted, through which all staff, including physicians, data collectors and analyzers, will be fully informed regarding the purpose and content of the study in an effort to standardize the electronic case report form (eCRF) completion process during data collection. In addition, the supervisors at the VRUH will offer guidance at each center when the first individual is enrolled, and the CRCTU-VRUH, which belongs to Carlos III Health Institute Platform for Support for Clinical Research (SCReN), will verify that the development of the clinical trial is carried out in accordance with the standards of good clinical practices and complies 100% with the current protocol. This team will conduct periodic on-site inspections to ensure a consistent and accurate study process.

### Statistical analysis

All statistical analyses were performed with R 4.2.3. All data were tested for normality, with Shapiro-Wilk test, and equality of variances through Levene test. The variables related to the baseline demographic and clinical data of the sample, as well as adverse events, will be analyzed descriptively. Continuous variables will be summarized using mean and standard deviation or median and interquartile range, while discrete variables will be reported in absolute value and percentage. To ensure group comparability, baseline sociodemographic and clinical characteristics were tested by 1-way ANOVA or Kruskal-Wallis tests for continuous variables or by chi-squared tests or Fisher’s exact test for qualitative variables.

Regarding comparative case vs control analyses, the main outcome variables will be analyzed using generalized linear mixed models (GLMM) following a Poisson distribution. Separate models will be conducted for each dependent variable, being case vs. control status considered the between-subject variable, and time (scheduled visits), the within-subject variable. The effects of status variable, time, and the interaction between them will be examined. Centers will be included as random effects in the model. All the models will include covariates such as sex and age. To build these models, adherence to the missing at random condition for the absent data must be met. For cross-sectional comparative analyses, multiple binary logistic regressions will be employed using the Wald method, which involves variable elimination through the backward method. The response variables will be recoded as 0 and 1 based on whether they surpass specific cutoffs, as determined by specialized clinical staff. Both intention-to-treat and per-protocol analyses will be performed.

We will employ Kaplan-Meier survival curves and log-rank tests to evaluate the time to all-cause medication discontinuation. Patient follow-up will commence from the study inclusion until discontinuation of the initial treatment or censoring. Survival time may be censored either at the end of the study period or due to loss to follow-up.

### Study progress and outlook

**Figure 1:**
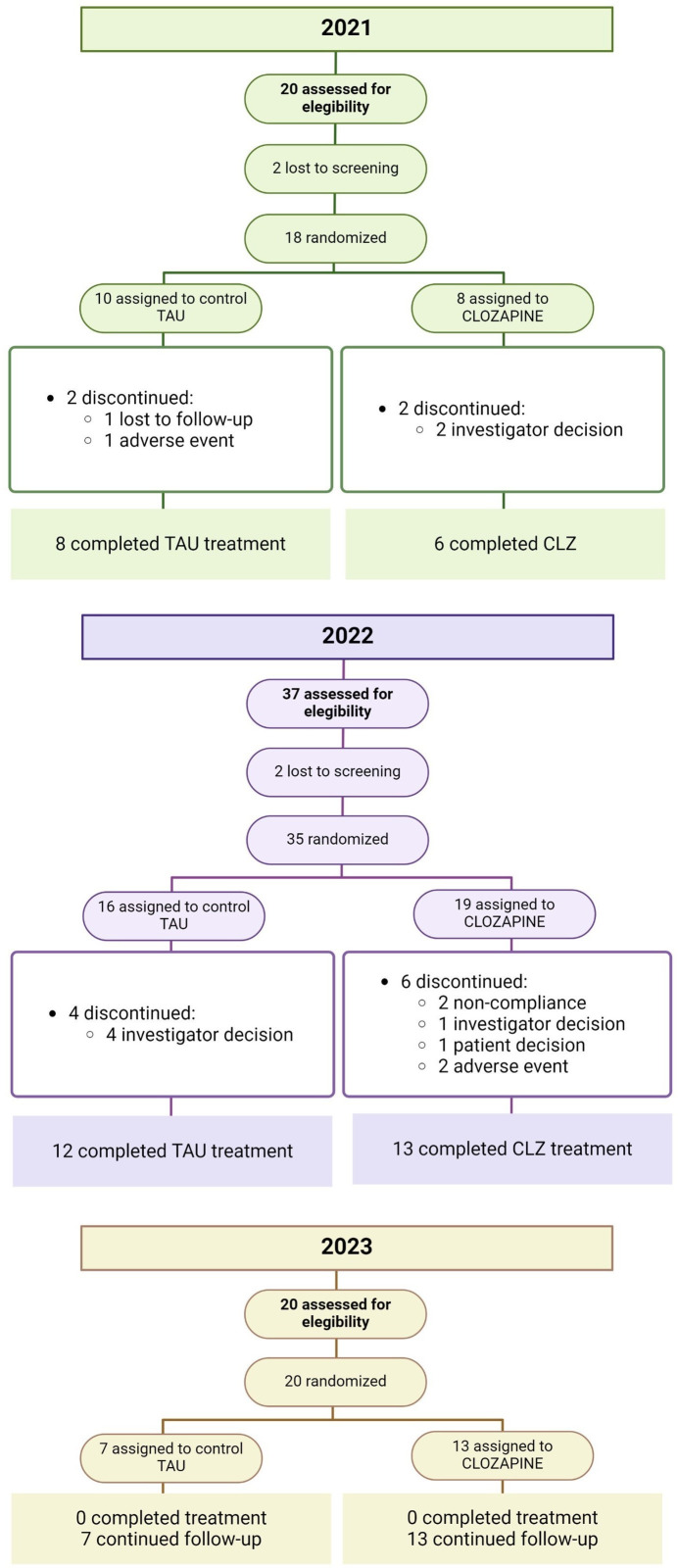
Overview of study flow and data. TAU, treatment as usual; CLZ, clozapine.

The study progress and individuals included in the different stages of the trial so far can be seen in **Figure 1**.

### Ethics approval and consent to participate

The study protocol was approved by the Ethics Committee for Drug Research of Seville, CEIm, (minutes no. 16/2020) and by the Spanish Agency of Medicines and Medical Devices, AEMPS, (EudraCT No. 2020-000091-37) in July 2020, authorizing the feasibility of the clinical trial. During the trial, the patient will take an authorized oral medication and will be monitored to ensure safety and efficacy. Before participating, the patient must sign an informed consent, agreeing to have their data reported in a database expressly designed for the study.

Carlos III Health Institute has funded the clinical trial that was authorized in 2019 call (ICI19/00026).

## Results

Seventy-seven individuals have been included in the study so far. The target population of the essay is a population of adults with intellectual disabilities and treatment-resistant psychotic disorders by definition, predominantly from a lower-middle-class background with access to the public healthcare system.

The research findings will be shared through publication in an international open-access peer-reviewed journal. This will comply with the provisions established in Royal Decree 1090/2015 of December 4, regulating Clinical Trials with Medicinal Products, the Ethics Committee for Research with Medicinal Products, and the Spanish Registry of Clinical Studies, Article 42. The principal investigator holds the responsibility of distributing these results to healthcare facilities.

## Discussion

This paper presents rationale for, and organization of CLOZAID protocol, which is an open-label, randomized, multicenter clinical trial to evaluate the efficacy and safety of clozapine in the treatment of resistant psychosis in individuals with ID. The results of clinical trials conducted in the general population may not be directly applicable to individuals with ID. This is the first clinical trial focused on the treatment of resistant psychosis conducted using a representative sample of the ID population.

Clozapine represents an optimal treatment for individuals with psychosis that present resistance to other pharmacological strategies (19, 20) as well as for comorbid conditions usually observed among individuals with ID (21–24). However, the use of clozapine in the treatment of resistant psychosis in ID is non-evidence based and rather scarce. Clozapine is nowadays underutilized in clinical practice given that it may involve severe side effects, what obligates clinicians to carry out a meticulous clinical follow up of in-treatment individuals involving, among others, the regular monitoring of neutrophils in peripheral blood (27–31). However, it is noteworthy that the rare occurrence of the cited side effects, coupled with the demonstrated efficacy of clozapine in treating resistant psychosis, presents a compelling case. Utilizing clozapine can help prevent the inefficient and risky overmedication that resistant individuals often face over the years. This underscores the significance of conducting a randomized and controlled clinical trial, such as the one outlined here, since the literature on the use of clozapine in resistant psychosis among ID individuals is limited to case series (35).

The findings from our study are expected to enhance our understanding of the effective and safe treatment of psychotic symptoms in individuals with ID. This knowledge may potentially catalyse a shift in the clinical management of this condition, ultimately leading to an improvement in the lives of these individuals worldwide and a reduction in the economic burden associated with treating comorbid psychosis in individuals with ID. We anticipate that the outcomes of our clinical trial will advocate for the use of clozapine as a beneficial treatment strategy for these individuals, potentially influencing a global shift in the clinical practice of managing psychosis in individuals with ID.

## Author contributions

MA-N: Writing – original draft, Writing – review & editing. BS-B: Data curation, Supervision, Writing – review & editing. PR-P: Data curation, Investigation, Writing – review & editing. LA-M: Investigation, Writing – review & editing. AG-G: Writing – review & editing. IR-G: Project administration, Writing – review & editing. NG-T: Investigation, Writing – review & editing. MR-V: Conceptualization, Writing – review & editing. SG-C: Writing – review & editing. CR-F: Investigation, Writing – review & editing. JV-M: Investigation, Writing – review & editing. FS: Investigation, Writing – review & editing. JC-B: Investigation, Writing – review & editing. RM-L: Investigation, Writing – review & editing. FM-C: Investigation, Writing – review & editing. BC-F: Funding acquisition, Resources, Supervision, Writing – review & editing.

## CLOZ-AID Group

Samuel Romero Guillena (Hospital Universitario Virgen Macarena), Álvaro López Díaz (Hospital Universitario Virgen Macarena), María Dolores Romero Lemos (Hospital Universitario Virgen del Rocío), María Conde Rivas (Hospital Universitario Virgen del Rocío), Ana Rubio García (Hospital Universitario Virgen del Rocío), Manuel Canal Rivero (Hospital Universitario Virgen del Rocío), Rubén Catalán Barragán (Hospital Universitario Virgen del Rocío), Irene Pans (Hospital Universitario Virgen del Rocío), María Luisa Gutierrez (Hospital Universitario Virgen del Rocío), Eduardo García Ramos-García (Hospital Universitario Virgen del Rocío), Ana Vilches (Hospital Universitario Virgen del Rocío), Beatriz Oda Plasencia (Hospital Universitario Virgen del Rocío), Ramón Terrón (Hospital Universitario Virgen del Rocío), Cristina Valdera (Hospital Universitario Virgen del Rocío), Manuela Rey (Hospital Universitario Virgen del Rocío), Demetrio Mármol (Hospital Universitario Virgen del Rocío), Cristina Esteban (Hospital Universitario Virgen del Rocío), Matilde Castaño (Hospital Universitario Virgen del Rocío), Juan Pedro Alcón (Hospital Universitario Virgen del Rocío), Nicolás Vucinovich (Hospital Universitario Virgen del Rocío), Luis R. Capitán (Hospital Universitario Virgen del Rocío), Cándido García (Hospital Universitario Virgen del Rocío), Matilde Blanco (Hospital Universitario Virgen de Valme), Álvaro J. Palma (AGS Osuna), Susana Herrera Caballero (AGS Jerez de la Fra., Costa Noroeste y Sierra de Cádiz), Asunta Torres Laborde (AGS Jerez de la Fra., Costa Noroeste y Sierra de Cádiz), Rocío Torrecilla Olavarrieta (AGS Jerez de la Fra., Costa Noroeste y Sierra de Cádiz), Melquíades Leon Macías (Hospital Regional Universitario de Málaga), Blanca García Montañes (Hospital Regional Universitario de Málaga), Juan Luis Prados Ojeda (AGS Córdoba), José Ángel Alcalá Partera (AGS Córdoba), Rafael Manuel Gordillo Urbano (AGS Córdoba), Laura Carrión Expósito (AGS Córdoba), Cristina Gómez Moreno (AGS Córdoba), Pablo Glez Domenech (Hospital Universitario San Cecilio), José Eduardo Muñoz Negro (Hospital Universitario San Cecilio), Ángeles Torres Prieto (Villablanca Serveis Assistencials), Annabel Folch Mas (Villablanca Serveis Assistencials), Juan José Mora Mesa (Hospital Virgen del Puerto), Rosa Mz Galindo San Valentín (Hospital Virgen del Puerto), Carlos Peña Salazar (Parc Sanitari Sant Joan de Deu), Ana Isabel Domínguez Panchón (Grupo Hermanas Hospitalarias), Cristina Irirte Iturria (Grupo Hermanas Hospitalarias), Paula Muñoz Hermoso (Grupo Hermanas Hospitalarias), David Gil Sanz (Grupo Hermanas Hospitalarias), Manuel Calvo Muñoz (Grupo Hermanas Hospitalarias), Georgia Denisa Simon (Grupo Hermanas Hospitalarias), Elena Rodríguez Cano (Grupo Hermanas Hospitalarias), Edith Pomarol Clotet (Grupo Hermanas Hospitalarias).
