# Supplementary material for: Efficacy of clozapine versus standard treatment in adult individuals with intellectual disability and treatment-resistant psychosis (CLOZAID): study protocol of a multicenter randomized clinical trial
Source: Front Psychiatry. 2024 May 14;15:1400621. doi: 10.3389/fpsyt.2024.1400621 (PMC11130499; doi:10.3389/fpsyt.2024.1400621)
Supplement: Supplementary file 1 [file Table_1.docx]

| **Supplementary Table 1. Clinical centers of the multicenter study** |
| --- |
| **Autonomous community: Andalucía** |
| Department of Psychiatry at the University Hospital Virgen del Rocio (Sevilla)  Department of Psychiatry at the University Hospital Virgen Macarena (Sevilla)  Department of Psychiatry at the Jerez University Hospital (Cádiz)  Virgen de la Caridad Residence for Severely Affected (Cádiz)  El Curtido Occupational Center (Cádiz)  University Regional Hospital (Málaga)  San Juan de Dios Care Center (Málaga)  Reina Sofía University Hospital (Córdoba)  Córdoba Sur Mental Health Unit (Códoba)  Montoro Mental Health Unit (Córdoba)  Montilla Mental Health Unit (Córdoba)  Cabra Mental Health Unit (Córdoba)  Andújar Mental Health Unit (Córdoba)  San Cecilio University Clinical Hospital (Granada)  La Purísima Foundation - Hermanas Hospitalarias (Granada) |
| **Autonomous community: Cataluña** |
| Villablanca Serveis Assistencials (Tarragona)  Benito Menni CASM in Sant Boi - Hermanas Hospitalarias (Barcelona)  Sant Joan de Déu Health Park (Barcelona)  FIDMAG Research Foundation (Barcelona) |
| **Autonomous community: Extremadura,** |
| Virgen del Puerto Hospital (Cáceres) |
| **Autonomous community: País Vasco** |
| Aita Menni Hospital in Mondragón - Hermanas Hospitalarias (Guipúzcoa) |
| **Autonomous community: Navarra** |
| Benito Menni Hospital Center in Elizondo - Hermanas Hospitalarias (Navarra)  Padre Menni Clinic in Pamplona - Hermanas Hospitalarias (Pamplona) |
| **Autonomous community: Cantabria** |
| Padre Menni Hospital Center in Santander - Hermanas Hospitalarias (Santander) |
| **Autonomous community: Madrid** |
| Benito Menni Care Complex in Arroyomolinos - Hermanas Hospitalarias (Madrid) |
